# Nitrogen Enrichment Reshapes Contrasting Microbial Networks in Northern Tibetan Alpine Meadow vs. Steppe

**DOI:** 10.3390/plants14172803

**Published:** 2025-09-07

**Authors:** Xueying Chen, Peili Shi, Jialuo Yu, Ge Hou, Ning Zong, Huixin Hei

**Affiliations:** 1 Institute of Geographic Sciences and Natural Resources Research, Chinese Academy of Sciences, Beijing 100101, China; 2 College of Resources and Environment, University of Chinese Academy of Sciences, Beijing 100190, China

**Keywords:** N addition, plant richness, pH, nutrient availability, microbial diversity, co-occurrence network, Northern Tibetan Plateau

## Abstract

Increased Nitrogen (N) input exerts significant impact on the functional integrity of terrestrial ecosystems, with alpine grasslands being particularly susceptible. Soil microbes are intricately intertwined with nearly all facets of essential biogeochemical cycle, underscoring their pivotal role in ecosystem processes. To elucidate how N enrichment modulates soil microbes and their diversity, 11-year N addition experiments were conducted in a semi-humid alpine meadow (AM) and an arid alpine steppe (AS) on the Northern Tibetan Plateau. We measured soil properties, aboveground net primary productivity (ANPP), plant diversity, microbial composition and diversity, as well as microbial co-occurrence networks. The results revealed that N additions profoundly reshaped microbial co-occurrence in alpine grasslands, albeit via divergent mechanisms in different ecosystems. In AM, N enrichment destabilized microbial networks mainly through reduced bacterial diversity linked to plant diversity loss. Conversely, in the harsher AS, N addition fostered closer microbial interactions, forming a more stable co-occurrence network despite lower plant richness, predominantly attributed to increased soil nutrient availability. Our results highlight the significance of co-occurrence networks as a key component of microbial biodiversity and emphasize the imperative of deciphering microbial interaction mechanisms to unravel soil functional dynamics under global nitrogen enrichment.

## 1. Introduction

Human activities such as the combustion of fossil fuels and the use of agricultural fertilizers have substantially increased nitrogen (N) inputs into terrestrial ecosystems [1,2]. Projections indicate that global N inputs may rise by 2.5 times or more by the end of this century [2,3]. This escalating N enrichment has been proven to pose severe threats to plant diversity, community structure, and ecosystem functioning [4], with particularly pronounced impacts in alpine grasslands. In most grassland ecosystems, N addition typically enhances aboveground net primary production (ANPP) by alleviating N limitation [5,6,7]; however, this increase is often accompanied by a decline in species richness. For instance, long-term N addition experiments demonstrated that even low-level N addition significantly reduced plant diversity, especially causing the loss of rare species [8]. Similarly, multi-level N addition studies in the Inner Mongolia and the Qinghai-Tibetan Plateau have shown that plant species diversity continues to decline progressively with increasing N application rates [9,10,11,12]. Moreover, N addition promotes the growth of annual species with rapid resource acquisition strategy [13], alters plant community dynamics [14] and exerts profound influences on the underground microbial world. Ecosystem function is thus closely linked to both plant community changes and soil microbial dynamics in response to N input [15].

Soil microbes, characterized by high diversity and complexity, are intricately linked to almost all facets of Earth’s essential biogeochemical cycle [16,17]. Through their roles in organic matter decomposition, nutrient cycling, soil structure formation, and plant productivity regulation [18,19], they act as key drivers of ecosystem diversity and functioning [20]. A growing body of research has explored microbial responses to N additions but inconsistencies remain. While some long-term N addition experiments reported minimal impacts on soil bacteria [21], meta-analyses have revealed an overall decline of soil bacterial biodiversity, as indicated by reduced Chao 1 and Shannon indices [22]. Fungal diversity responses are more variable, showing either a decrease [22] or no significant change [23], likely due to fungi’s stronger resistance to environmental stress compared to bacteria. Beyond α-diversity, N additions also alter microbial community composition [24]. Continuous N inputs increase soil N availability, induce soil acidification [25], and modify plant biomass and community composition [14], which in turn affect bacterial [26] and fungal community structure [27]. Notably microbial responses are not solely driven by direct effects; microbial community composition is dynamic and closely linked to species changes [20,28]. Plants influence microbes through multiple pathways: first, increased plant residue inputs provide more substrates, stimulating microbial abundancy and activity [29]; second, the altered litter chemical composition diminishes the competitive advantage of oligotrophic taxa while favors copiotrophic taxa with readily available carbon sources [30]. Most studies assume plants as primary determinants of soil microbial community composition [31,32,33]. Given to the pivotal underground ecological processes in ecosystem functioning, there is an urgent need to clarify the mechanisms underlying microbial diversity and community to N additions, particularly in different plants diversity.

A critical yet often overlooked dimension of soil biodiversity is the complex network of interactions among microbial taxa, alongside species richness and abundance [34,35]. In recent years, molecular ecological network analysis has become widely adopted in soil microbial ecology [36]. These ecological relationships can be visualized as co-occurrence networks, where microbial taxa serve as nodes [37], and edges represent interactions within the biological community [36]. Network topological features, including average degree (connectivity) and modularity (community compartmentalization), characterize the intensity of microbial associations and their functional roles [38]. It is generally accepted that the complexity of a network’s structure is directly related to the number of nodes and edges. The increased complexity is believed to enhance the network’s resilience to interference. Co-occurrence network analysis has been applied across diverse ecosystems, including farmlands, grasslands [39,40], and permafrost [41], to explore potential microbial interactions. Microbial co-occurrence networks are sensitive to environmental factors: N addition-induced changes in soil nutrient supply and pH can alter microbial interactions, thereby reshaping network structure [35]. Additionally, plant community variation profoundly influences interactions of microbial taxa and microbial networks through modifications in root architecture, litter inputs, microenvironmental conditions, resources availability and symbiotic relationships [42,43,44]. However, microbial network responses to N additions vary markedly across ecosystems, and the underlying mechanisms remain poorly understood. In this study, we defined network stability as the resistance and resilience of microbial interactions to N addition, complexity as the overall size and connectivity of the network (e.g., the number of nodes, edges, and average degree), and modularity as the degree of compartmentalization of the network into relatively independent modules. These distinctions allow a more precise interpretation of microbial responses under N addition.

The Qinghai-Tibetan Plateau, as the highest and largest plateau on Earth, exhibiting extremely sensitive to human-induced global and environmental changes [45]. In this study, we selected two types of grasslands that cover the largest area of the Northern Tibetan Plateau, a semi-humid diverse-species alpine meadow (AM) in the east and an arid poor-species alpine steppe (AS) in the west. The two ecosystems differ significantly in soil nutrient status and plant composition, leading to different microbial communities. After N addition, soil microbes in these two ecosystems may exhibit different interactions, thus providing an ideal natural platform to study microbial responses. Leveraging our 11-year long-term nutrient addition experiments in these distinct grasslands, this study aimed to: (1) investigate changes in soil microbial community composition and diversity and key influencing factors; (2) examine the soil microbial interactions after N addition under different grassland types.

## 2. Results

### 2.1. Changes in Soil Properties and Plant Characteristics

**Figure 1:**
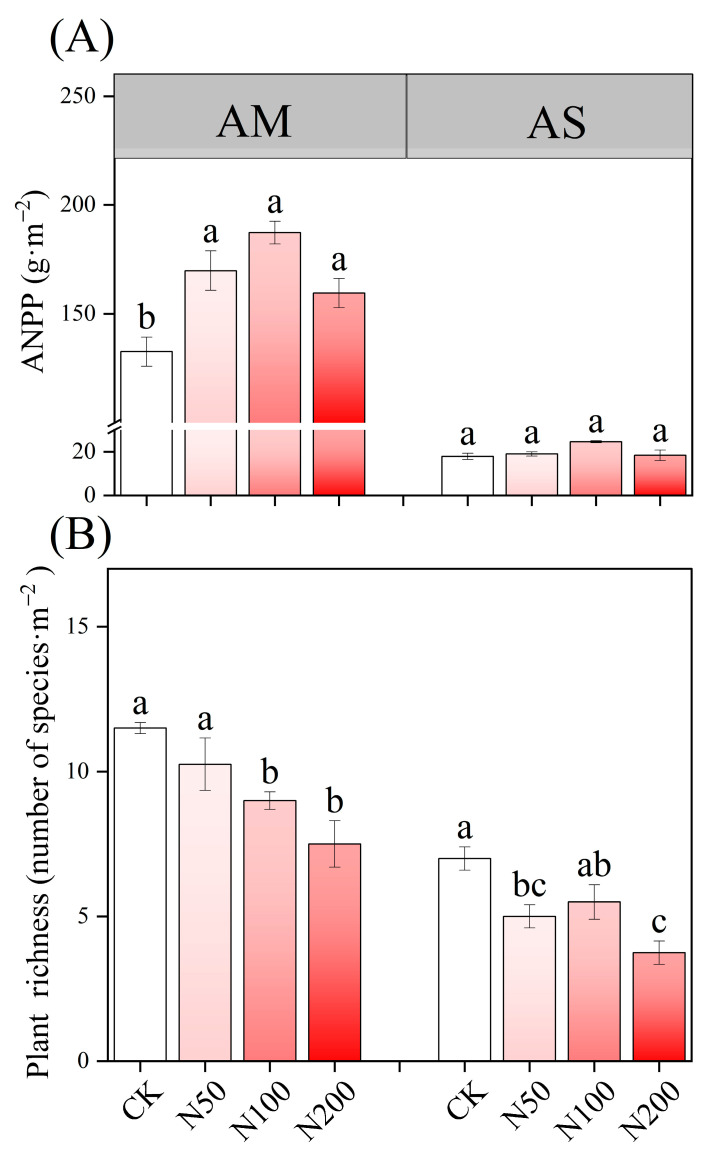
Effects of N additions on plant ANPP (**A**) and species richness (**B**) in AM and AS. Different lowercase letters represent significant differences among treatments in the same grass-land at the 0.05 level.

N additions exerted a significant effect on soil acidification in AM, accompanied by an increase in TAN content; however, no significant effects were observed on TN and TP. In contrast, soil pH in AS remained neutral without significant change. Under higher N addition, TN, TP, and AP in AS increased significantly and TAN demonstrated a significant increase as well (Table 1). N additions significantly increased ANPP in AM (Figure 1A), but led to a decreased in species richness in both AM and AS (Figure 1B).

### 2.2. Soil Microbial Community Composition and Diversity

**Figure 2:**
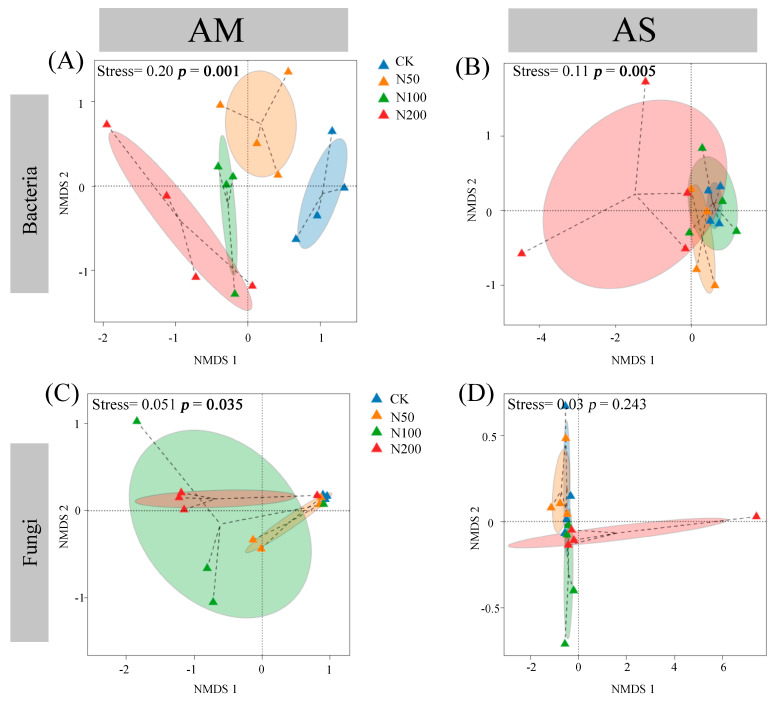
Nonmetric multidimensional scaling based on Bray–Curtis distance of bacterial in AM (**A**) and AS (**B**), and fungal in AM (**C**) and AS (**D**).

**Figure 3:**
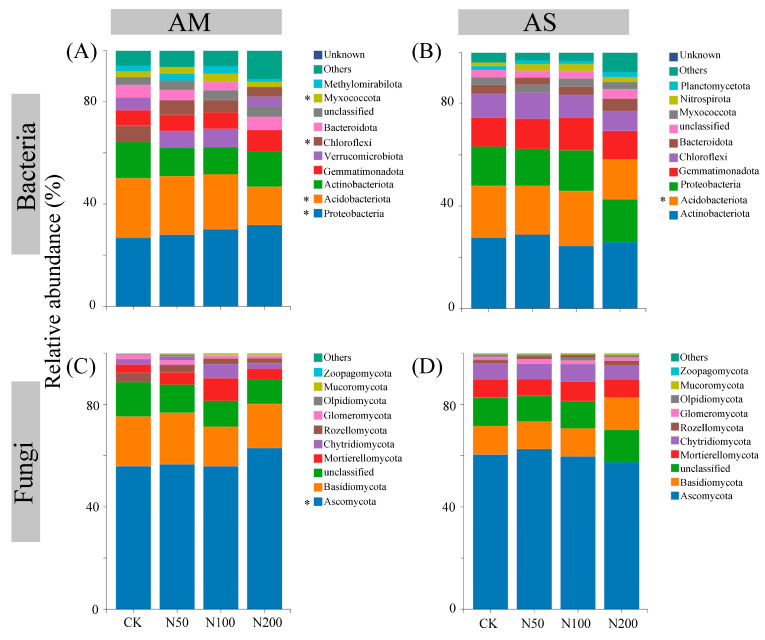
Differences in the top ten taxa at phylum level for soil bacteria in AM (**A**) and AS (**B**), fungi in AM (**C**) and AS (**D**). Significance level: * *p* < 0.05.

The rarefaction curves show clear asymptotes, indicating that sampling covered the majority of distinct ASVs (Figure S1). Quality control analysis generated 863,231 and 891,890 sequences in AM and AS for bacteria, which were clustered into 36,505 and 35,134 ASVs, respectively. For fungi, quality control analysis generated 759,614 and 1,069,110 sequences in AM and AS, which were clustered into 29,336 and 27,699 ASVs, respectively. Soil bacterial community compositions significantly changed under N additions in both grasslands, as shown by NMDS combined with ANOSIM similarity analysis (Figure 2A,B). In contrast, significant shifts in soil fungal community were only observed in AM (Figure 2C,D). Based on the differences in the top ten taxa at the phylum level, the relative abundances of Proteobacteria, Myxococcota, and Ascomycota significantly increased in AM, while those of Acidobacteriota and Chloroflexi decreased (Figure 3A,C). In AS, N additions led to a significant reduction in the relative abundance of Acidobacteriota (Figure 3B). With increasing N additions, the bacteria diversity in both grasslands and fungi diversity in AM significantly decreased (Table 2).

### 2.3. Soil Microbial Co-Occurrence Network

**Figure 4:**
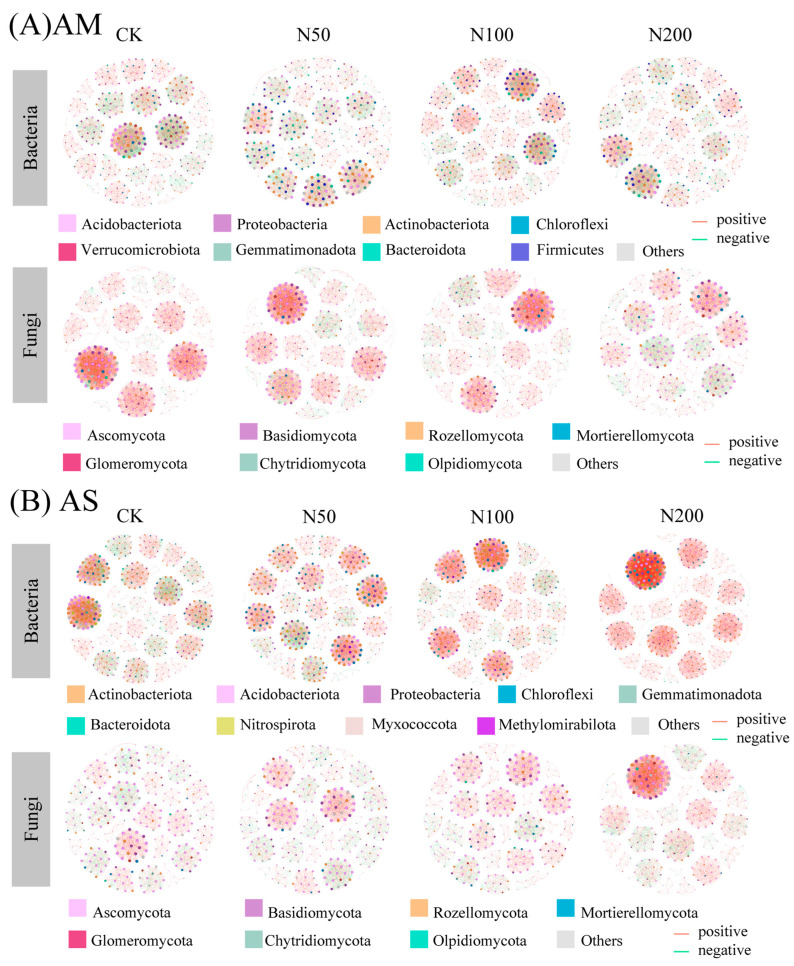
Co-occurrence network of bacteria and fungi communities in AM (**A**) and AS (**B**) at ASVs level. Red and green lined indicated significant positive and negative connections (|Spearman correlation| > 0.5, *p* < 0.05), respectively. The size of each node is proportional to the number of connections (i.e., degree). The thickness of the edges is proportional to the Spearman’s correlation coefficient. The network is colored by phylum.

Responses of soil bacterial and fungal co-occurrence network revealed that N additions induced distinct shifts in microbial network patterns. In AM, compared with CK (356 nodes, 2960 edges in bacterial and 294 nodes, 3062 edges in fungal), N additions resulted in simplified bacterial and fungal co-occurrence networks, as evidenced by reduced numbers of nodes, edges, average degree, and network density, whereas network modularity increased (Figure 4A and Table 3).

In AS, the number of bacterial nodes showed a decreasing trend with increasing N input, declining from 431 in CK to 394 in N200. Notably, the number of edges increased significantly in the N200, primarily driven by a sharp rise in positive edges despite a drastic decline in negative edges. Consequently, the average degree of bacterial nodes increased significantly under N200 (21.85 vs. 27.98), indicating a more tightly connected network. Meanwhile, network density increased slightly from 0.05 to 0.07, and modularity declined from 0.90 to 0.78, suggesting a less compartmentalized network. In contrast, fungal networks in AS exhibited an increase in both node and edge numbers. Positive and negative edges showed opposite trends: positive edges increased while negative edges decreased. Average degree and density of fungal networks also increased. Consistent with bacterial networks, fungal network modularity decreased from 0.94 in CK to 0.80 in N200 (Figure 4B and Table 3).

### 2.4. Environment Factors Affecting the Structure of Soil Microbial Community and Co-Occurrence Networks

**Figure 5:**
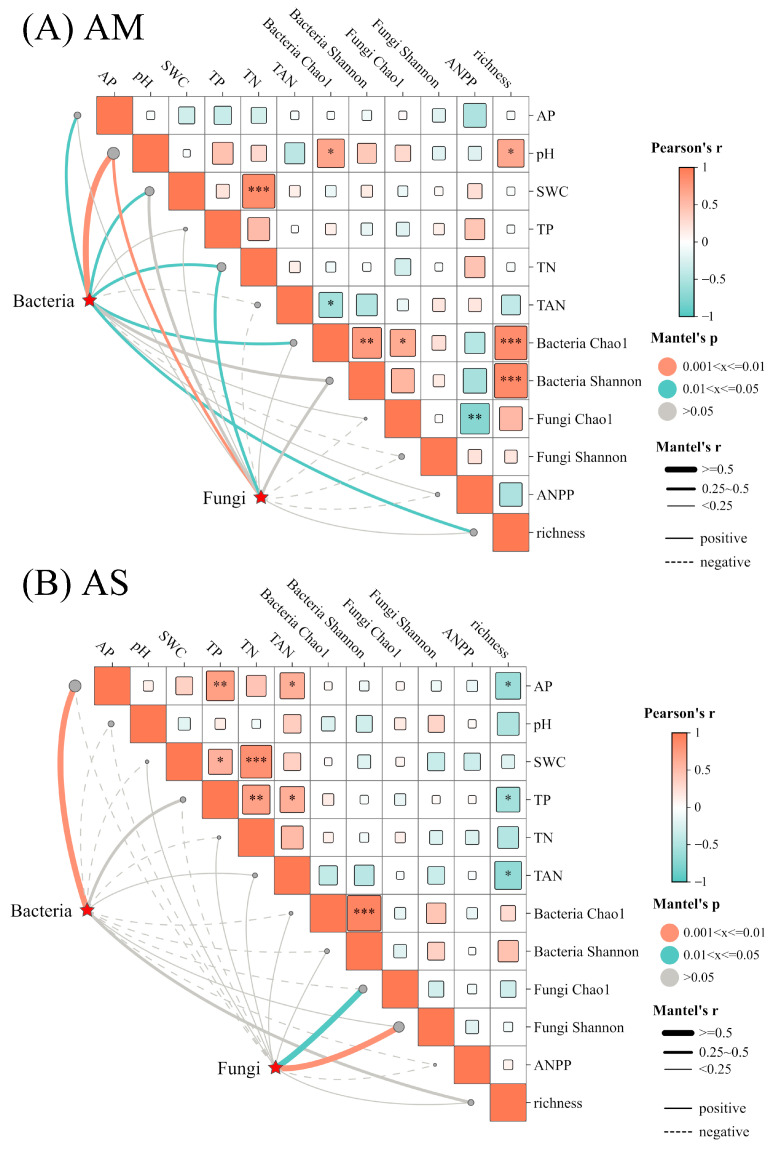
Mantel’s correlations between soil bacterial, fungal community composition, and environmental factors. The solid and dashed lines indicate positive and negative correlations, respectively. The size of squares is indicative of the absolute values of the correlation coefficient. *, **, *** represent *p* < 0.05, *p* < 0.01, and *p* < 0.001, respectively.

Mantel-test results revealed that in AM, soil bacterial and fungal community composition were significantly associated with soil pH and TN. Additionally, soil AP, SWC, and plant richness showed significantly positive correlation with bacterial community composition. In AS, soil bacterial communities are mainly associated with soil AP (Figure 5A,B). However, ANPP exhibited no impacts on microbial community composition in both types of grasslands.

**Figure 6:**
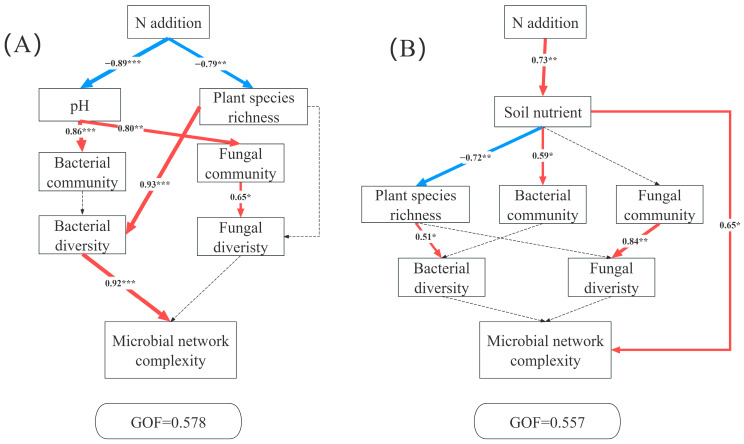
Partial least squares path modeling analysis (PLS-PM) showing the direct and indirect effects of N additions on microbial network complexity in AM (**A**) and AS (**B**). Soil nutrient (TP, TN, AP, TAN), the bacterial community (the relative abundance of bacteria at the phylum level), the fungal community (the relative abundance of fungi at the phylum level), bacterial diversity (Chao 1 and Shannon index of bacteria), fungal diversity (Chao 1 and Shannon index of fungi), microbial network complexity (nodes, edges, positive edges, negative edges, average degree, density, and modularity). The red and blue arrows represent positive and negative pathways, respectively, and solid and dashed lines show significant and non-significant pathways, respectively. The thickness of the arrows indicated the strength of the relationship. Significance level: * *p* < 0.05, ** *p* < 0.01, *** *p* < 0.001.

Structural equation models (SEM) revealed distinct pathways through which N additions influenced microbial network complexity in the AM and AS. In AM, the complexity of co-occurrence network was driven jointly by plants and microbes. N addition significantly decreased soil pH and richness. The reduced pH strongly affects bacterial community and fungal community structures. Plant species richness exhibited a significant positive relation with bacterial diversity, thus leading to a significant change in microbial network complexity (Figure 6A). By contrast, the primary pathway linking N additions to microbial network complexity in AS operated through soil nutrients (Figure 6B).

## 3. Discussion

### 3.1. Soil Microbial Community Composition and Diversity and Its Determinants Under N Additions

Proteobacteria, Acidobacteriota, and Actinobacteriota were identified as the dominant bacterial taxa in both grasslands (Figure 3), consistent with findings from studies in Northeastern China [46], Arctic [47], and other alpine regions [48]. This suggests that specific taxa within these phyla have adapted to cold habitats [46]. Similarly, the most abundant fungal taxa, Ascomycota and Basidiomycota have been reported in other grassland ecosystems [49], indicating broad ecological adaptability. NMDS analysis demonstrated that both bacterial and fungal communities in AM underwent significant changes following N additions. In contrast, in AS, only bacterial communities exhibited significant changes. The observed community changes align with the copiotrophic hypothesis, characterized by increased relative abundances of copiotrophic phyla and decreased abundances of oligotrophic phyla [50]. The Proteobacteria phylum in bacteria and Ascomycota phylum in fungi, typically recognized as copiotrophic taxa, follow a r-Strategy with rapid growth and short life cycles [21,50], while oligotrophic phyla such as Acidobacteriota and Chloroflexi exhibit K-Strategy [50].

Our study demonstrated that N additions significantly reduced the Chao 1 and Shannon indices of soil bacterial in both grasslands, in line with observations in other ecosystems [51,52,53]. The responses of bacteria communities to high N inputs strongly depends on soil background nutrient availability or acidification status [24]. It is reported that soil bacterial diversity is closely associated with soil pH, even in large-scale analysis [54]. In AM, the significant decrease in pH, coupled with the narrow range of optimal pH for bacterial growth, was the primary driver of reduced bacterial abundance [24,54]. However, in AS, where pH remained stable. In addition to the buffering effects of metal ions, the low temperature and precipitation in AS may slow down urea hydrolysis and migration, leading to minor pH decline. The relative higher baseline pH in AS also mitigated the N-induced pH decline, and the decline in bacterial diversity was mainly due to increased nutrient availability. Furthermore, the greater reduction in the Chao 1 index compared to the Shannon index suggests that rare species were more vulnerable to N additions, as the Chao 1 is particularly sensitive to rare species loss [55], while the Shannon index integrates both species abundance and evenness [56]. Consequently, under N addition, the greater decline in Chao 1 index suggested that rare species might have disappeared more [22]. Unlike bacteria, the fungi exhibited weaker correlation with soil pH, supporting previous findings that fungi are less sensitive to pH fluctuations than bacteria [57]. In AM, the decreased Chao 1 index with stable Shannon index indicated that fungal diversity loss was predominantly driven by rare species disappearance. Moreover, fungal communities also showed weaker shifts than bacteria, which may explain why fungal diversity remained unchanged in AS despite N additions.

### 3.2. Changes in Co-Occurrence Network Patterns and Topological Characteristics

Microbial interactions play a pivotal role in shaping community structure and function [38]. Microbial co-occurrence networks provide a visualized framework to explore microbial interactions [58]. Network complexity can be represented by topological metrics such as node number, edge number (positive or negative), average degree, density, and modularity [59]. In our study, we found that in AM, bacterial network under N100 exhibited the highest number of node count, edge number, and average degree, but higher N addition (i.e., N200) increased modularity, weaken network connectivity, and reduced network stability [60]. Notably, the gradual decrease in negative edges in bacterial network with N additions suggests that elevated nutrient levels alleviated resistance or competitive relationships, shifting microbial interactions toward functional similarity [61]. For AM fungal communities, N additions reduced node number, positive edges, and average degree, while increasing negative edges and modularity. This pattern aligns with resource competition theory: when microbial taxa share similar resource requirements or ecological niches, resource scarcity intensifies interspecific resource competition, manifested by increased negative correlations [61,62]. Such competitive and cooperative interactions may enhance community performance by improving resource utilization efficiency [63]. Fungal responses typically lag behind bacterial dynamics, and N addition may disrupt nutrient symbiotic relationships, further altering network structure [64,65].

In contrast, AS exhibited distinct network responses to N additions. Both bacterial and fungal co-occurrence networks showed increased edge numbers, dominated by positive correlations, indicating synergistic interactions that collectively respond to environmental changes [66], consistent with findings from Qinghai-Tibetan Plateau [35]. Elevated average degree and reduced modularity in AS networks suggest increased complexity and tighter connectivity. This pattern may arise from two mechanisms: first, N addition-induced nutrient enrichment promoted cooperative resource conversion efficiency [67]; second, high N stress drove microbial associations to enhancing environmental adaptability, thereby stabilizing system [68].

### 3.3. Mechanisms Underlying Changes in Co-Occurrence Network Structure of Different Grasslands Under N Additions

Our SEM revealed contrasting mechanisms driving microbial network complexity in AM and AS under N additions (Figure 6), highlighting ecosystem-specific mediation pathways. In AM, N additions reduced soil pH and plant diversity. Plant diversity directly regulated bacterial diversity, which in turn influenced network complexity. N addition-induced acidification is a well-demonstrated phenomenon [25,69,70,71], and prior research showed that while N alleviates plants N limitation, it exacerbates limitations by P, light, and micronutrient limitations [72]. According to resource competition exclusion theory, N-induced convergence of limiting resources reduces ecological niche dimensions, intensifying interspecific competition and decreasing plant richness [72,73]. Furthermore, we found that plant diversity significantly regulated bacterial diversity. Reduced plant diversity likely diminished the diversity of organic substrates, resources and carbon compounds available for soil microbes [74], weakening the positive plant-bacterial diversity relationship [74]. In AM, bacterial communities exerted a greater influence on network complexity than the fungi, leading to reduced network stability under N additions. In AS, N additions significantly increased soil nutrient availability. Despite nutrient-induced changes in plant diversity and bacterial communities, network complexity was primarily driven by enhanced nutrient availability [67]. Compared to AM, AS’s lower precipitation creates harsher environment conditions for microbial survival [35]. This stress may select for cooperative microbial interactions to adapt to environmental changes, resulting in tighter network connectivity and increased complexity.

This study reveals ecosystem-specific mechanisms underlying microbial network responses to nitrogen additions, emphasizing that acidification-driven plant-bacteria interactions dominate in AM, while nutrient-mediated cooperative interactions prevail in AS. The contrasting patterns challenge the generality of nitrogen-induced microbial network simplification, highlighting the role of environmental context (e.g., aridity, soil buffering capacity) in shaping microbial responses. These findings advance our understanding of how grassland microbial communities adapt to nitrogen enrichment through network reconfiguration, with implications for predicting ecosystem stability under global nitrogen deposition scenarios. Although network indicators provided valuable insights into microbial interactions, their functional impacts on ecosystem processes such as carbon cycling still require further assessment. Further research will focus on microbial functions, identify functional genes associated with carbon cycling, and elucidate the microbial mechanisms governing these cycles under nutrient additions.

## 4. Materials and Methods

### 4.1. Study Area and Experiment Design

The study was conducted on the Northern Tibetan Plateau, with an average altitude of above 4500 m. The regional climate is classified as alpine continental climate, with mean annual temperature (MAT) below 0 °C. The coldest month occurs in January and the warmest in July. While the east–west variation in MAT is less than 2 °C, the mean annual precipitation (MAP) decreases significantly from east to west, ranging from 430 mm to 280 mm. This heterogeneous precipitation pattern has shaped distinct grassland types across the Northern Tibetan Plateau: wetter alpine meadow (AM) in the east, dominated by *Korbresia pygmea*, *Stipa purpurea*, and arid alpine steppe (AS) in the west, dominated by *S. purpurea*, *Astragalus confertus*. Our AM and AS sampling sites are located in Nagqu (31°34′ N, 92°34′ E) and Nyima (32°18′ N, 87°23′ E), respectively. Herbaceous plants were categorized into four main functional groups, i.e., grasses, sedges, legumes, and forbs.

N addition experiments were initiated in 2013 and continued for eleven years until 2024. Based on local background of N deposition, four different N additions were established at each site, namely control treatment (CK), three N addition levels (50, 100, and 200 kg N hm^−1^ year^−1^, hereafter referred to as N50, N100, and N200, respectively). The experiment adopted a random block design with four replications (4 m × 4 m quadrats) per treatment, with adjacent quadrats spaced two meters apart. N was applied annually as urea (CO(NH_2_)_2_) during the early growing season in mid-June.

### 4.2. Plant Community and Soil Sampling

The plant community surveys were conducted annually during the peak biomass of alpine plants, usually in the middle of August. The biomass in the sampled peak season was considered the aboveground net primary productivity (ANPP). Specifically, a 1 m × 1 m quadrat was randomly selected within each plot, avoiding overlap with the previous year’s survey area to ensure independence in species count. A 0.5 m× 0.5 m sub-quadrat was then established at the center of each quadrat for aboveground biomass sampling. Plants within this sub-quadrat were harvested, sorted by species, and oven-dried at 65 °C to constant weight for biomass determination. The sum biomass of all species was defined as the aboveground net primary productivity (ANPP) of the community.

Subsequent to the community survey, soil samples were collected from each quadrat by combining three soil cores (0–20 cm depth) obtained using a soil drill. All soil samples were passed through a 2 mm sieve to remove stones, litter, and roots, then divided into three subsamples: one preserved as fresh soil for available nutrient analysis, the second air-dried for total nutrient determination, and the third stored in liquid nitrogen for high-throughput microbial sequencing.

### 4.3. Soil Properties

Soil pH was measured using a glass electrode meter in a 2.5:1 water-soil suspension after shaking for 30 min. Soil water content (SWC) was determined via the gravimetric method after drying at 105 °C for 24 h. Soil total nitrogen (TN) was analyzed using the Kjeldahl method. Soil NH_4_^+^-N and NO_3_^−^-N were extracted from 5 g fresh soil with 20 mL of 2 M KCL solution, and quantified using a continuous flow analyzer. NH_4_^+^-N and NO_3_^−^-N is summed as total available N (TAN). Soil total phosphorus (TP) was measured with an ultraviolet spectrophotometer. Soil available phosphorus (AP) was determined by the molybdenum blue method at a wavelength of 700 nm. Soil organic carbon (SOC) was analyzed using the potassium dichromate wet oxidation-redox titration method.

### 4.4. Soil Microbial Community and Diversity

Soil total DNA was extracted from 0.5 g fresh soil using the TGuide S96 Magnetic Soil/Stool DNA kit (Tiangen Biotech, Beijing, Co., Ltd., Beijing, China). Bacterial 16S rRNA bacterial genes (V3-V4 region) were amplified using primers 338F (5′-ACTCCTACGGGAGGCAGCA-3′) and 806R (5′GGACTACHVGGGTWTCTAAT-3′). For fungal identification, the internal transcribed spacer (ITS) region was amplified with primer ITS1F (5′-CTTGGTCATTTAGAGGAAGTAA-3′) and ITS2 (5′-GCTGCGTTCTTCATCGATGC-3′). The amplified products were sequenced at high-throughput sequencing on the Illumina Novaseq 6000 platform. Raw sequences were quality-filtered using Trimmomatic software (v0.33), followed by quality control, denoising, splicing, and de-chimerism of all raw sequences using the DADA2 plug-in in QIIME to obtain ASVs. Bacterial 16S rRNA and fungal ITS sequences were taxonomically classified and annotated against Silva 138 and UNITE 8.0 databases, respectively. α-diversity was evaluated using the Shannon and Chao 1 index. β-diversity was assessed via non-metric multidimensional scaling (NMDS) based on the Bray–Curtis distances.

### 4.5. Microbial Co-Occurrence Network

We constructed co-occurrence networks to examine whether N addition altered soil microbial co-occurrence patterns. First, bacteria and fungi ASVs with a relative abundance greater than 0.05% were selected. Spearman’s correlation coefficients between these ASVs were calculated using the *corr.test*() function in the ‘psych’ package in R (version 4.03). To reduce the false positive results, the ‘BH’ correction was used to adjust the correlation *p* values. Then correlation coefficient |r| > 0.5 and adjusted *p* < 0.05 were retained co-occurrence network construction. Finally, Gephi (version 0.10.1) was used to visualize bacterial and fungal co-occurrence networks and calculate topological properties, including the number of nodes, edges, positive edges, and negative edges, averaged degree, density and modularity.

### 4.6. Statistical Analysis

SPSS (version 22.0) was used for the data analysis. Values are given as mean ± standard error (n = 4). A one-way ANOVA was used to identify the effects of N addition on soil properties, ANPP, and plant richness, with Duncan’s test for pairwise comparisons at *p* < 0.05. The rank sum test was used to analyze differences in soil microbial relative abundance. Partial least squares path modeling (PLS-PM) was used to evaluate the possible pathways through which N addition affects microbial network complexity. Prior to PLS-SEM construction, Mantel-tests were conducted to screen for significant factors included in the model. Goodness of fit (GOF) was used to evaluate model fitness. All graphs were drawn using Origin 2025 software.

## 5. Conclusions

The 11-year nutrient enrichment experiments revealed that N additions profoundly reshaped soil microbial co-occurrence patterns in Northern Tibetan Plateau grasslands, with divergent mechanisms between AM and AS. In AM, N addition significantly altered both bacterial and fungal community composition and reduced diversity. In contrast, AS exhibited significant changes exclusively in bacterial communities and diversity, whereas fungal communities remained largely unchanged. N additions led to less stable bacterial and fungal networks in AM, characterized by weakened connectivity. This destabilization was primarily driven by reduced bacterial diversity, which itself resulted from decreasing plant diversity. Conversely, in the harsher AS, N additions promoted closer microbial interactions, forming more stable co-occurrence networks despite lower plant richness, driven by increased soil nutrient availability. These findings contribute to a more comprehensive understanding of the divergent microbial network responses to N enrichment and highlighting habit-specific mechanisms governing microbial regulation of co-occurrence networks, addressing critical gaps in long-term nutrient enrichment research in high-altitude regions. Our results suggest that moderate N addition (<50 kg N hm^−2^ year^−1^) had relatively limited impacts on microbial diversity in AM and AS, whereas higher N addition significantly reduce both plant and bacterial richness. Therefore, to preserve biodiversity and maintain microbial-mediated ecosystem functions, N inputs should be careful controlled. This study provides a robust foundation for predicting the functional responses of alpine grasslands to N enrichment, informing targeted management strategies, i.e., preserving plant diversity in AM and regulating nutrient dynamics in AS to sustain microbial-mediated ecosystem functions.

## Figures and Tables

**Table 1:** Effects of N additions on soil properties in AM and AS.

Soil Properties	Grassland Types	Treatments				
		CK	N50	N100	N200	
pH	AM	5.92 ± 0.01 a	5.45 ± 0.12 b		5.63 ± 0.01 b	4.93 ± 0.03 c
	AS	7.54 ± 0.4 a	7.65 ± 0.0 a		8.02 ± 0.03 a	7.91 ± 0.07 a
AP (mg·kg^−1^)	AM	4.30 ± 0.4 a	3.12 ± 0.12 a		3.34 ± 0.4 a	4.23 ± 0.4 a
	AS	2.27 ± 0.2 b	5.93 ± 0.3 ab		3.20 ± 0.3 b	9.77 ± 0.3 a
TP (g·kg^−1^)	AM	0.45 ± 0.0 ab	0.45 ± 0.01 ab		0.47 ± 0.0 a	0.43 ± 0.0 b
	AS	0.29 ± 0.0 b	0.29 ± 0.0 b		0.35 ± 0.0 a	0.35 ± 0.0 a
TN (g·kg^−1^)	AM	2.74 ± 0.2 a	2.65 ± 0.07 a		3.27 ± 0.1 a	2.49 ± 0.3 a
	AS	1.21 ± 0.0 b	1.51 ± 0.0 a		1.16 ± 0.1 b	1.50 ± 0.1 a
TAN (mg·kg^−1^)	AM	19.81 ± 4.4 b	24.68 ± 0.7 ab		29.09 ± 0.4 a	29.81 ± 2.4 a
	AS	9.37 ± 1.3 c	44.18 ± 4.2 b		44.06 ± 4.8 b	81.36 ± 1.2 a

Note: Values are given as mean ± standard error (n = 4). Different lowercase letters represent significant differences among treatments in the same grassland at the 0.05 level. AM: alpine meadow. AS: alpine steppe. AP: available P. TP: total P. TN: total N. TAN: total available N, the sum of NH_4_^+^-N and NO_3_^−^-N.

**Table 2:** Effects of N additions on soil microbial α-diversity in AM and AS.

		Grassland Types	CK	N50	N100	N200
Bacteria	Chao 1	AM	2721.90 ± 95.9 a	2272.88 ± 182.6 ab	2351.03 ± 322.4 ab	1800.66 ± 87.7 b
		AS	2567.75 ± 116.4 a	2386.46 ± 208.8 ab	1878.26 ± 250.0 b	1989.5 ± 177.5 b
	Shannon	AM	10.14 ± 0.1 a	9.93 ± 0.1 ab	9.77 ± 0.2 ab	9.63 ± 0.1 b
		AS	10.09 ± 0.0 a	9.87 ± 0.1 ab	9.63 ± 0.1 b	9.67 ± 0.1 b
Fungi	Chao1	AM	2195.34 ± 119.6 a	2007.88 ± 14.6 ab	1760.39 ± 80.3 b	1810.70 ± 105.4 b
		AS	1849.24 ± 01.1 a	1723.18 ± 19.7 a	1780.11 ± 58.4 a	1902.03 ± 96.1 a
	Shannon	AM	8.51 ± 0.1 a	8.65 ± 0.1 a	8.15 ± 0.3 a	8.45 ± 0.1 a
		AS	8.74 ± 0.1 a	8.62 ± 0.0 a	8.70 ± 0.1 a	8.60 ± 0.2 a

Note: Values are given as mean ± standard error (n = 4). Different lowercase letters represent significant differences among treatments in the same grassland at the 0.05 level. AM: alpine meadow. AS: alpine steppe.

**Table 3:** Changes in network topological parameters of the microbial under N additions in AM and AS.

		Topological Features	CK	N50	N100	N200
AM	Bacteria	Node number	356	331	394	356
		Edge number	2960	2627	3499	2667
		Positive edge	1923	1691	2645	1885
		Negative edge	1037	936	854	782
		Average degree	16.63	15.87	17.76	14.98
		Density	0.05	0.05	0.05	0.04
		Modularity	0.90	0.92	0.91	0.92
	Fungi	Node number	294	303	287	288
		Edge number	3062	2939	2429	2112
		Positive edge	2950	2636	2184	1662
		Negative edge	112	303	245	450
		Average degree	20.83	19.40	16.93	14.67
		Density	0.07	0.06	0.06	0.05
		Modularity	0.80	0.86	0.82	0.90
AS	Bacteria	Node number	431	421	412	394
		Edge number	4708	4259	4248	5512
		Positive edge	3289	3513	3766	5472
		Negative edge	1419	746	482	40
		Average degree	21.85	20.23	20.62	27.98
		Density	0.05	0.05	0.05	0.07
		Modularity	0.90	0.92	0.89	0.78
	Fungi	Node number	281	293	285	286
		Edge number	1599	1844	1817	2545
		Positive edge	1184	1382	1562	2198
		Negative edge	415	462	255	347
		Average degree	11.38	12.59	12.75	17.80
		Density	0.04	0.04	0.04	0.06
		Modularity	0.94	0.92	0.92	0.80

## Data Availability

Data will be made available on request.

## Supplementary Materials

The following supporting information can be downloaded at https://www.mdpi.com/article/10.3390/plants14172803/s1: Figure S1: The rarefaction curves of bacteria and fungi in AM and AS.
