# Supplementary figures and images for: Nitrogen Enrichment Reshapes Contrasting Microbial Networks in Northern Tibetan Alpine Meadow vs. Steppe
Source: Plants (Basel). 2025 Sep 7;14(17):2803. doi: 10.3390/plants14172803 (PMC12430419; doi:10.3390/plants14172803)

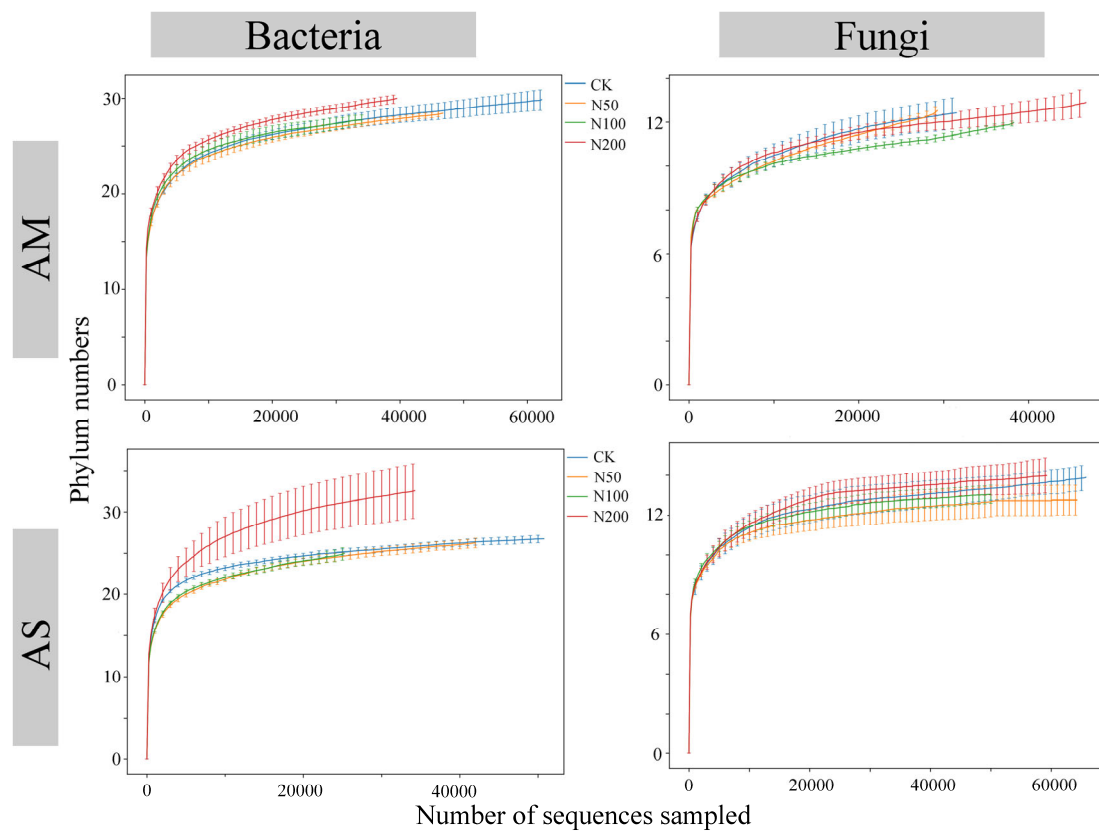

Figure S1 The rarefaction curves of bacteria and fungi in AM and AS

Supplement: Supplementary file 1 [file plants-14-02803-s001.zip › plants-3828754-supplementary.pdf]
